# AI medical device post-market surveillance regulations: consensus recommendations by the European Society of Radiology

**DOI:** 10.1186/s13244-025-02146-8

**Published:** 2025-12-12

**Authors:** Renato Cuocolo, Diana Bernardini, Daniel Pinto dos Santos, Michail E. Klontzas, Tugba Akinci D’Antonoli, Luís Curvo Semedo, Robin Decoster, Merel Huisman, Elmar Kotter, Luis Martí-Bonmatí, Costin Minoiu, Emanuele Neri, Konstantin Nikolaou, Maija Radzina, Evis Sala, Susan C. Shelmerdine, Laurens Topff, Michelle C. Williams, European Society of Radiology (ESR)

**Affiliations:** 1 Department of Medicine, Surgery, and Dentistry, University of Salerno, Baronissi, Italy; 2 European Society of Radiology (ESR), Vienna, Austria; 3 Department of Radiology, University Medical Center, Mainz, Germany; 4 Artificial Intelligence and Translational Imaging (ATI) Lab, Department of Radiology, School of Medicine, University of Crete, Heraklion, Crete, Greece; 5 Department of Diagnostic and Interventional Neuroradiology, University Hospital Basel, Basel, Switzerland; 6 Department of Pediatric Radiology, University Children’s Hospital Basel, Basel, Switzerland; 7 Local Health Unit—Aveiro Region; Faculty of Medicine, University of Coimbra, Coimbra, Portugal; 8 Odisee UoAS, Brussels, Belgium; 9 Radboud University Medical Center, Department of Radiology and Nuclear Medicine, Nijmegen, The Netherlands; 10 Department of Diagnostic and Interventional Radiology, Medical Center—University of Freiburg, Freiburg, Germany; 11 Medical Imaging Department and Biomedical Imaging Research Group at Hospital Universitario y Politécnico La Fe and Health Research Institute, Valencia, Spain; 12 University of Medicine “Carol Davila”, Bucharest, Romania; 13 Department of Translational Research, University of Pisa, Pisa, Italy; 14 Department of Diagnostic and Interventional Radiology, University Hospital of Tuebingen, Tuebingen, Germany; 15 Radiology Department, Riga Stradins University, Riga, Latvia; 16 Dipartimento di Scienze Radiologiche ed Ematologiche, Universita Cattolica del Sacro Cuore and Dipartimento Diagnostica per Immagini e Radioterapia Oncologica, Policlinico Universitario A. Gemelli IRCCS, Rome, Italy; 17 Great Ormond Street Hospital, London, UK; 18 University College London (UCL), London, UK; 19 Department of Radiology, Netherlands Cancer Institute, Amsterdam, Netherlands; 20 British Heart Foundation Centre for Research Excellence, University of Edinburgh, Edinburgh, United Kingdom

**Keywords:** Artificial intelligence, Regulation, Implementation, AI literacy, Human oversight

## Abstract

**Abstract:**

The increasing integration of artificial intelligence as medical devices (AIaMDs) within diagnostic imaging necessitates a robust understanding of associated regulatory frameworks among clinical practitioners. Despite the growing commercial availability and adoption of AIaMD, a significant awareness gap persists among radiologists regarding pertinent European Union regulations, including the Medical Device Regulation (MDR) and the novel EU AI Act, both of which lack explicit provisions tailored to AI components. This regulatory ambiguity underscores a critical need for clarified guidelines concerning “high-risk” AI classification and best practices for safe deployment within the radiological workflow. Legal responsibility for AIaMD Post-Market Surveillance (PMS) primarily rests with software providers, yet radiologists are expected to contribute to the ongoing monitoring of safety and performance. Recognizing the need to raise awareness and provide practical guidance, the European Society of Radiology (ESR) eHealth and Informatics Subcommittee, supported by the ESR AI Working Group, conducted a modified Delphi procedure involving 16 domain experts (of which 14 acted as panelists) to establish a set of shared recommendations. These aim to establish essential practices for AIaMD PMS and post-market clinical feedback (PMCF), as stipulated by the MDR and partially updated by the AI Act. This paper also provides an overview of relevant regulations to enhance awareness among all stakeholders, particularly deployers (e.g., radiologists) and providers (e.g., vendors). These recommendations represent a foundational step towards improving consistency in AIaMD deployment, providing a critical reference standard for physicians navigating the unique challenges posed by these novel technologies.

**Critical relevance statement:**

Radiologists need to familiarize themselves with AIaMD EU regulations due to shared PMS responsibilities and current ambiguities. ESR recommendations aim to bridge this awareness gap, standardizing safe AI deployment and enhancing clinical feedback within medical imaging.

**Key Points:**

- Radiologists need a clear understanding of EU regulations for AIaMDs, as current laws lack imaging-specific guidance.
- There is a shared responsibility for AIaMD safety, with radiologists contributing to PMS and clinical feedback systems.
- The ESR provides crucial recommendations to standardize AI deployment and improve clinical feedback in imaging.

**Graphical Abstract:**

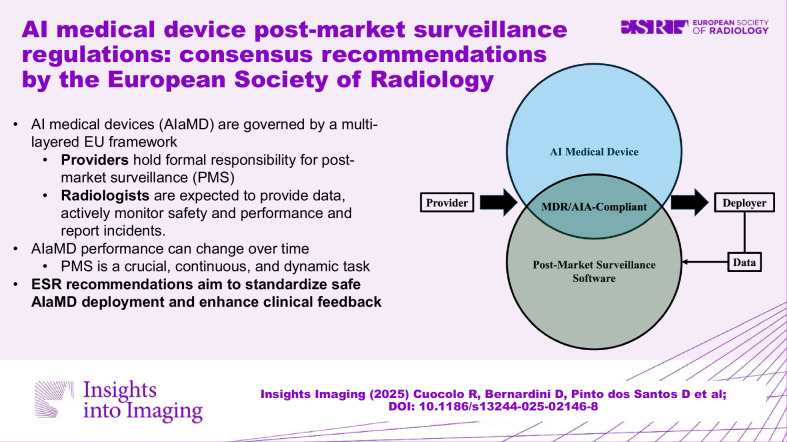

## Introduction

The past decade has seen an increase in Artificial Intelligence as medical devices (AIaMD) within the imaging domain entering the commercial market, available for clinical deployment. Despite the associated increase in AIaMD adoption, a significant majority of radiologists are unfamiliar with appropriate EU regulations surrounding their use. A recent survey conducted by the ESR showed only 29% of AIaMD deployers (and 10% of responders not using AIaMDs) consider themselves familiar with medical device regulation (MDR) and post-market surveillance (PMS) [1]. This lack of awareness of AIaMD regulations can be partially justified by their complexity, as the MDR was not originally tailored to AI components, and does not present specific requirements to account for AIaMD use. However, a more recent regulatory policy, the EU AI Act, is not necessarily any clearer, and its novelty may also explain the lack of widespread awareness amongst radiologists [2]. Hopefully, incoming guidelines will clarify the requirements for “high-risk” AI classification, concerning the software to be used within the radiological workflow, as well as the best practices to be followed to ensure their safe deployment.

As the use of AI tools in medical imaging becomes increasingly widespread, the need for shared guidelines and clearly established best practices is more critical than ever. Moreover, although the legal responsibility for PMS lies with the software provider, radiologists are expected to play a role in it, namely, the ongoing monitoring of the safety and performance of these tools after deployment in clinical practice. In this context, the European Society of Radiology (ESR) eHealth and Informatics Subcommittee, with the support of the ESR AI Working Group, has conducted a modified Delphi procedure to establish recommendations on the complex topic of AI medical device PMS and post-market clinical feedback (PMCF), and the resulting statements are presented in this paper. These are essential practices for the safe use of AI medical devices, as established by the MDR and partially updated by the AI Act. The following text also includes a brief overview of the relevant regulations to raise awareness in all stakeholders, especially deployers (e.g., radiologists using AIaMD tools) and providers (e.g., AIaMD vendors), within medical imaging.

## Regulations in the EU

The regulation of post-market monitoring of AI-based medical devices in the European Union is determined by a multi-layered legal framework that combines the horizontal application of rules on AI, with sectoral legislation on medical devices [3].

Two instruments from the regulatory framework are particularly relevant:

- The MDR (EU) 2017/745 (MDR) governs the safety, performance, and clinical oversight of medical devices.
- The AI Act (Regulation (EU) 2024/1689), which entered into force in August 2024, introduces horizontal obligations on all AI systems, including those used in healthcare.

**Fig. 1:**
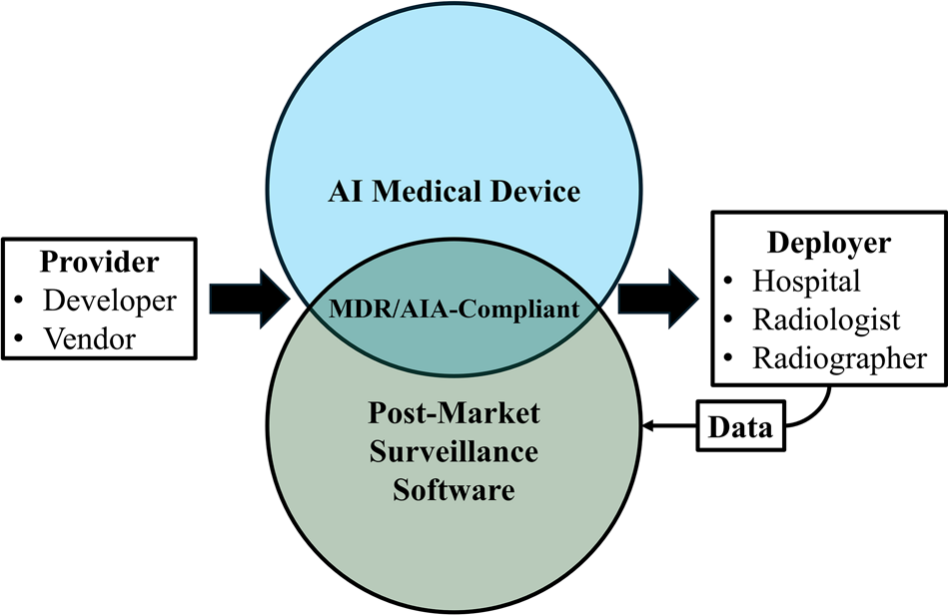
Diagram summarizing the interplay between AI as a medical device provider, deployers, and PMS regulations in the European Union

Together, these sets of rules aim to have continuous compliance, safety, and trust throughout the life of the AIaMD through core obligations of PMS and PMCF. In addition to the MDR, which defines PMS and PMCF criteria, the AI Act provides additional stipulations for “high-risk” medical devices. In the context of healthcare, this includes AI systems that are themselves medical devices or function as a safety component of devices regulated by the MDR and subject to third-party conformity assessment [Article 6(1) and Annex I AI Act]. To facilitate the approach to these topics, a glossary of relevant terms is made available in Table 1, while Fig. 1 provides a schematic representation of the involved actors.

**Table 1:** Glossary of terms related to AI medical device PMS and clinical feedback regulations

Term	Explanation
Deployer	AI Act, Article 3(4) defines ‘Deployer’ as any natural or legal person, public authority, agency, or other body using an AI system under its authority, except where the AI system is used in the course of a personal non-professional activity. In a healthcare setting, deployers are hospitals, radiology departments, or individual clinicians who use an AI system during diagnosis or treatment. They have specific legal duties under the AI Act, including ensuring human oversight, transparency, monitoring, and incident reporting obligations.
Provider	AI Act, Article 3(2) defines ‘Provider’ as a natural or legal person, public authority, agency, or other body that develops an AI system or that has an AI system developed and places it on the market or puts it into service under its own name or trademark, whether for payment or free of charge. Additionally, MDR, Article 2(30) provides the comparable definition of ‘Manufacturer’ as the natural or legal person who manufactures or fully refurbishes a device or has a device designed, manufactured, or fully refurbished, and markets that device under its name or trademark. The provider or manufacturer is typically the AI software vendor or developer (e.g., a medtech company or platform operator). They are responsible for complying with PMS and PMCF obligations and must maintain regulatory oversight throughout the product’s lifecycle.
PMS	As defined in Article 2(60) of the MDR, PMS refers to “all activities carried out by manufacturers in cooperation with other economic operators to institute and keep up to date a systematic procedure to proactively collect and review experience gained from devices they place on the market […]”. PMS is a continuous process to ensure that a medical device, including AI systems used in radiology, remains safe, performs as intended, and complies with relevant regulatory requirements over time.
PMCF	Defined in Article 2(61) and detailed in Annex XIV Part B of the MDR, PMCF involves the proactive collection of clinical data to confirm safety and performance and identify unknown risks or long-term effects. In other words, PMCF is a specific form of PMS focused on gathering clinical evidence from real-world use to support ongoing validation and update risk assessments.

### PMS

PMS refers to the range of activities and tasks conducted after the launch of an AIaMD into the market to ensure its long-term safety, effectiveness, and performance in real-world clinical settings.

Under Article 2(60) MDR, PMS encompasses “all activities carried out by manufacturers in cooperation with other economic operators to institute and keep up to date a systematic procedure to proactively collect and review experience gained from devices they place on the market.” Therefore, PMS is not a one-time evaluation, but rather a longitudinal safety and performance assessment across the AI device’s lifecycle [4].

In AI-enabled radiological devices (including software), PMS is crucial to identify performance decay or data drifts, latent clinical risks (e.g., missed lesions in cancer diagnostics), subgroup underperformance, as well as usability challenges when integrated into radiological workflows [5].

Under Article 83 of the MDR, manufacturers must establish and maintain a PMS system for every medical device, proportional to its risk class and appropriate to its type. The system must gather, record, and assess pertinent information on the quality, performance, and safety of the device during its life cycle actively and systematically. Annex III Part B details the methods for gathering user feedback, identifying trends, and evaluating the effectiveness of corrective actions. Ultimately, the outcomes of the PMS activities are used to update the device’s risk management document, clinical evaluation, labeling, and, where necessary, start corrective actions.

It is evident from the wording of the MDR that the manufacturer or provider of the medical device holds formal responsibility for PMS tasks. Under Article 87 MDR, they are required to report serious incidents and field safety corrective actions, while Article 88 MDR mandates them to detect patterns of non-serious incidents through trend reporting activities—an obligation that might also be triggered by direct reporting by clinicians. Lastly, manufacturers must investigate serious incidents promptly and in coordination with national competent authorities under Article 89 MDR.

In the context of AI-enabled medical devices deployed for image analysis, lesion detection, or workflow triage, PMS serves the key purpose of ensuring that these systems maintain safety, effectiveness, and clinical relevance over time. Due to the peculiarity of machine learning models trained on historical data, the performance of AI-enabled medical devices might change over time due to factors such as population shifts, interoperability upgrades, or evolving clinical practice. Therefore, unlike traditional medical devices, AI tools can exhibit data drift or performance degradation, should they are not be properly monitored [3]. It is therefore up to the PMS to accomplish the challenging but crucial task of monitoring predictive accuracy and clinical relevance over time.

This need for dynamic and continuous oversight is reinforced by Article 72 of the AI Act, mandating that AI providers implement a PMS system specifically tailored to AI. This system must actively and systematically collect and analyze data on the AI system’s performance, enabling providers to verify ongoing compliance with legal obligations on robustness, transparency, and risk mitigation.

However, the AI Act emphasizes the importance of deployers in the post-market ecosystem. While providers retain primary responsibility and liability, deployers are expected to use AI systems as instructed, assign appropriate human oversight functions, ensure the logging and quality of input data under their control, monitor outputs for unexpected behaviors, and report relevant incidents [Articles 12, 26(2), 26(5), and 26(6) AI Act] [2]. Additionally, according to Article 26(6) of the AI Act, deployers must retain system logs for at least six months to support traceability, audits, and incident reporting. Clinicians’ feedback is instrumental for both reactive incident handling, as well as proactive continuous system monitoring [6]. Identifying the clear roles of AI providers and deployers for a successful interoperable PMS framework—taking into account real-world usage conditions (i.e., interaction with other clinical software and workflow components)—is essential to ensure the continued safety, effectiveness, and trustworthiness of AIaMDs.

### Post-market clinical follow-up (PMCF)

A specific element of PMS under the MDR is PMCF, which is detailed in Article 2(61) together with Annex XIV Part B. PMCF requires manufacturers (i.e., providers under the AI Act) to continuously collect and evaluate clinical data to confirm the safety and performance under actual clinical conditions.

Through PMCF tasks, manufacturers should be able to identify previously unknown risks or long-term side effects of the launched medical devices. Furthermore, by collecting and evaluating clinical data, the real-world diagnostic accuracy and benefit-risk analysis of the devices can be updated and confirmed.

According to Article 83(3) MDR, data gathered through both PMS and PMCF should be used to improve the design and usability of medical devices, identify trends, detect emerging risks, and inform the need for any corrective actions.

## ESR recommendations

### Development process

A modified Delphi procedure was employed to develop the statements included in this article. An expert panel constituted by 2 contributors with medical imaging and regulatory knowledge (R.C. and D.B.) prepared an initial list of recommendations regarding PMS and PMCF implementation in medical imaging, focused on AI medical devices and the interplay of the MDR and AI Act. These were designed to provide clear indications to physicians and other healthcare professionals involved in the medical imaging community, who are currently using an AIaMD in practice or planning to do so in the future. A secondary aim was to provide expert opinion to the EU regulatory bodies in regard to the practical implications of AIaMD use under current conditions.

Experts were then contacted to provide feedback through an online form on the resulting statements. An invitation was sent to members of the ESR eHealth and Informatics Subcommittee and ESR AI Working Group, due to their domain knowledge. A minimum of 10 participants was considered to be required. All participants expressed their consent to participate before voting. The consensus threshold was set at 75%, based on a 5-point Likert scale. Specifically, “agreement” (Likert score = 4) and “strong agreement” (Likert score = 5) votes were considered as favorable for the inclusion of the statement, for the purpose of reaching a consensus. Items that did not reach consensus were revised, accounting for comments made during their assessment. The possibility of proposing new items was also presented to participants at this time. Subsequently, only the revised and new items were presented to the expert group for reassessment.

Finally, items reaching consensus after the two rounds were included in the present manuscript.

**Fig. 2:**
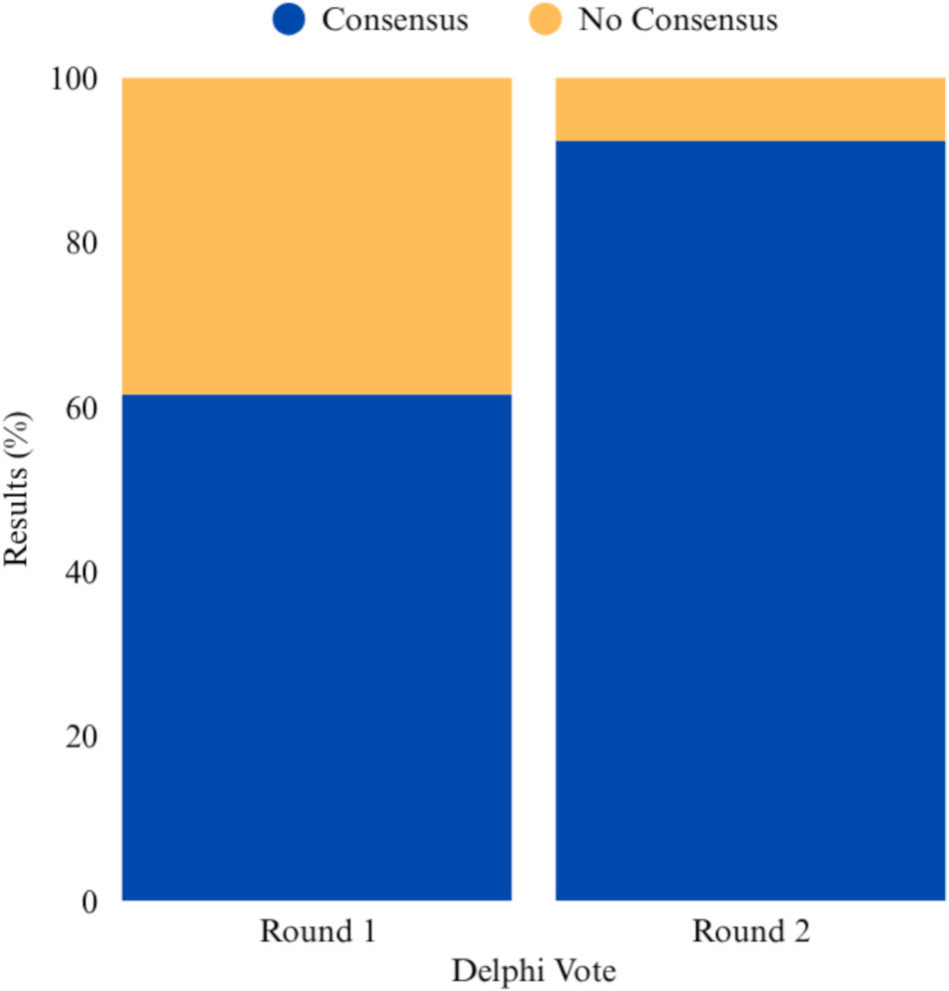
Stacked bar plot showing the proportion of items achieving consensus during each Delphi round

The initial draft included a total of 13 items. Out of 16 invited experts, 14 (88%) were able to participate and complete both Delphi rounds. After the first round, 8/13 (62%) of the items reached a consensus for their inclusion. No new items were proposed. After revision, the 5 remaining items were re-assessed, leading to 12 achieving the required consensus threshold. Details of the Delphi voting results and items evaluated in each round are presented in the supplementary materials and Fig.2.

The resulting recommendations are presented in the following text, accompanied by a brief commentary, and summarized in Table 2.

**Table 2:** Statements by the ESR regarding the implementation of PMS and clinical feedback systems for AI medical devices in clinical imaging

Number	Statement
1	Medical imaging professionals are not sufficiently familiar with PMS and clinical follow-up requirements and regulations pertaining to AI medical devices in clinical practice, including in regards to their duties as deployers of such systems.
2	The PMS should include a platform to ensure accessibility of continuously collected performance data to deployers, allowing both monitoring by medical imaging practitioners and ad hoc reporting of relevant events, as required by PMCF.
3	The data collection, which may be performed by deployers of AI medical devices, should be implemented at an institutional level (e.g., through a semi-automated system managed by dedicated personnel within a department/hospital) rather than relying on voluntary action by single physicians.
4	All deployers of a given AI medical device should be able to request access to aggregate data regarding PMS and system performance, in a form compliant with current data protection regulations.
5	Upon deployment of a new AI medical device, the PMS system must be made available at the same time. If a new function is added to a pre-existing device, the PMS must be contextually updated to incorporate any new information necessary.
6	Beyond continuous monitoring, periodic reviews (e.g., every 6–12 months) of PMS data must be performed by providers and presented to deployers, to allow informed ongoing use of the medical device and facilitate timely detection of performance degradation.
7	Some use cases (e.g., image reconstruction) may require the collection of additional data (e.g., non-AI reconstructed images) to allow periodic performance assessment of the AI medical device.
8	To allow effective PMS, providers must provide the AI medical device baseline accuracy metrics, including uncertainty measures (e.g., 95% confidence intervals). These should be clearly visible on the PMS platform to deployers in order to facilitate the detection of underperformance or other issues.
9	Deployers of AI medical devices should consider informing the referring clinician and/or patients about AI-related issues and their resolution, if warranted by the gravity and/or clinical relevance of the event (e.g., an incident worthy of reporting according to PMCF regulations).
10	To facilitate compliance by deployers with PMCF duties, it would be preferable for the platform employed for PMS to also allow user feedback to be recorded. This information should also be accessible to other physicians within the same institution, through the same platform, to facilitate local awareness of critical issues with an AI medical device.
11	The ESR advocates for interoperable PMS standards, easing the use of shared platforms for AI medical devices from multiple providers. This practice would greatly increase the accessibility and manageability of PMS by providers and deployers alike, as the number of AI medical devices and their adoption in clinical practice is expected to increase over the years.
12	Providers may employ shared software platforms to optimize access to multiple AI medical devices. In this case, the use of interoperable PMS standards is recommended to facilitate the aggregation of data from all AI medical devices. For example, access through a unified user interface at the software platform level to monitor all devices delivered through said platform would be preferable rather than siloed PMS systems within each medical device’s dedicated interface.

### Statements

**Statement 1:** Medical imaging professionals are not sufficiently familiar with PMS and clinical follow-up requirements and regulations pertaining to AI medical devices in clinical practice, including regarding their duties as deployers of such systems.

**Comment:** A recent survey by the ESR and European Society of Medical Imaging Informatics highlighted the lack of awareness by radiologists regarding AI regulations and PMS specifically, independently of their current status as deployers of AI medical devices or not [1].

**Statement 2:** The PMS should include a platform to ensure accessibility of continuously collected performance data to deployers, allowing both monitoring by medical imaging practitioners and ad hoc reporting of relevant events, as required by PMCF.

**Comment**: Data collection alone is not sufficient to allow medical imaging practitioners to be fully aware of the ongoing performance (and potential degradation) of AI medical devices deployed at an institutional level. This information is clearly essential to meet some of the obligations of deployers, such as PMCF.

**Statement 3:** The data collection, which may be performed by deployers of AI medical devices, should be implemented at an institutional level (e.g., through a semi-automated system managed by dedicated personnel within a department/hospital) rather than relying on voluntary action by single physicians.

**Comment:** Currently, some providers of AI medical devices rely on periodic reporting by deployers on a voluntary basis of data regarding AI performance in clinical practice. These practices should be substituted by systematic data collection leveraging institutional resources (e.g., IT infrastructure) to ensure reliability and a more robust collection of evidence.

**Statement 4:** All deployers of a given AI medical device should be able to request access to aggregate data regarding PMS and system performance, in a form compliant with current data protection regulations.

**Comment:** The accessibility of data on the performance of AI medical devices could represent a significant aid for physicians in assessing the reliability of the system and recognizing issues that may negatively affect patient care on time.

**Statement 5:** Upon deployment of a new AI medical device, the PMS system must be made available at the same time. If a new function is added to a pre-existing device, the PMS must be contextually updated to incorporate any new information necessary.

**Comment:** Given the critical nature of PMS, no delay is acceptable in implementing the data collection and performance monitoring tools that should accompany all AI medical devices in clinical use.

**Statement 6:** Beyond continuous monitoring, periodic reviews (e.g., every 6–12 months) of PMS data must be performed by providers and presented to deployers, to allow informed ongoing use of the medical device and facilitate timely detection of performance degradation.

**Comment:** A periodic summary report, with variable timing based on how critical the medical device is concerning patient care, represents a necessary tool to ensure the feasibility of performance review by deployers, even without in-depth technical knowledge.

**Statement 7:** Some use cases (e.g., image reconstruction) may require the collection of additional data (e.g., non-AI reconstructed images) to allow periodic performance assessment of the AI medical device.

**Comment:** Given the wide range of tasks for which AI medical devices may be employed, a ground truth to be used for performance and quality assessment may not always be available. If possible, the necessary data has to be periodically collected to allow such assessments to be made. AI-enhanced image reconstruction represents a typical example of such a situation, as non-AI reconstructed images are not acquired in clinical routine to serve as a reference standard.

**Statement 8:** To allow effective PMS, providers must provide the AI medical device baseline accuracy metrics, including uncertainty measures (e.g., 95% confidence intervals). These should be clearly visible on the PMS platform to deployers in order to facilitate the detection of underperformance or other issues.

**Comment:** As a baseline, performance has to be established for certification of AI medical devices, this data should be made available to deployers to ease the task of performance monitoring for PMS and PMCF purposes, avoiding unnecessary delays in detecting degradations in performance after clinical deployment.

**Statement 9:** Deployers of AI medical devices should consider informing the referring clinician and/or patients about AI-related issues and their resolution, if warranted by the gravity and/or clinical relevance of the event (e.g., an incident worthy of reporting according to PMCF regulations).

**Comment:** Given the novelty of AI medical devices and the different levels of familiarity with their use across medical specialties and for patients, best practices on how to manage issues in AI medical device performance and limit their impact on patient management need to be evaluated on a case-by-case basis. Communication with the other stakeholders involved in patient care is paramount to reducing the risk of negative consequences.

**Statement 10:** To facilitate compliance by deployers to PMCF duties, it would be preferable for the platform employed for PMS to also allow user feedback to be recorded. This information should also be accessible to other physicians within the same institution, through the same platform, to facilitate local awareness of critical issues with an AI medical device.

**Comment:** As PMCF represents a distinct task from PMS, the reporting of adverse events should be streamlined as much as possible, leveraging the technologies already available for PMS purposes. Availability of such data to other deployers (in accordance with data protection laws) is also essential to allow rapid adjustment to compensate for AIaMD issues.

**Statement 11:** The ESR advocates for interoperable PMS standards, easing the use of shared platforms for AI medical devices from multiple providers. This practice would greatly increase the accessibility and manageability of PMS by providers and deployers alike, as the number of AI medical devices and their adoption in clinical practice is expected to increase over the years.

**Comment:** To reduce friction between AI medical device use and PMS, common standards would greatly benefit the interoperability and ease of access to the necessary information, reducing the burden on deployers when interacting with multiple devices on a regular basis.

**Statement 12:** Providers may employ shared software platforms to optimize access to multiple AI medical devices. In this case, the use of interoperable PMS standards is recommended to facilitate the aggregation of data from all AI medical devices. For example, access through a unified user interface at the software platform level to monitor all devices delivered through said platform would be preferable rather than siloed PMS systems within each medical device’s dedicated interface.

**Comment:** If a shared medical device delivery platform is employed, centralizing PMS on a single platform may represent a significant advantage in removing barriers to deployers in accessing critical PMS data when monitoring the performance of the AI medical devices delivered through the shared software platform.

## Conclusion

Medical devices using AI technology are becoming increasingly common in clinical practice. A gap remains in terms of awareness by medical imaging practitioners, as well as established best practices, as available in other domains of radiology. This situation is particularly apparent in the context of PMS and PMCF, where they are clearly established in principle by active current regulations, but not enacted upon, given unclear standardization and requirements at the moment. The recommendations made by the ESR in this regard hopefully represent a first step towards establishing the necessary guidelines and awareness amongst our community to improve consistency in the deployment and monitoring of AI as medical devices, while also allowing physicians to have a clear reference standard when approaching these novel technologies, which present unique challenges compared to previous practice.

## Supplementary information

ELECTRONIC SUPPLEMENTARY MATERIAL

## Abbreviations

AIaMDs: Artificial intelligence as medical devices
ESR: European Society of Radiology
MDR: Medical device regulation
PMCF: Post-market clinical feedback
PMS: Post-market surveillance
