# Supplementary material for: AI medical device post-market surveillance regulations: consensus recommendations by the European Society of Radiology
Source: Insights Imaging. 2025 Dec 12;16:275. doi: 10.1186/s13244-025-02146-8 (PMC12701188; doi:10.1186/s13244-025-02146-8)
Supplement: Supplementary file 1 — ELECTRONIC SUPPLEMENTARY MATERIAL [file 13244_2025_2146_MOESM1_ESM.pdf]

AI Medical Device Post-Market Surveillance Regulations: Consensus Recommendations by the European Society of Radiology

ELECTRONIC SUPPLEMENTARY MATERIAL

Delphi Round 1

Statement: Medical imaging practitioners are not sufficiently familiar with post-market surveillance (PMS) and clinical follow-up requirements and regulations pertaining to AI medical devices in clinical practice, including in regards to their duties as deployers of such systems.

Votes (100% in favor, achieved consensus)

Likert	Votes (n)
1	0
2	0
3	0
4	4
5	10

Statement: The responsibility of PMS lies exclusively on the AI medical device developer and provider. Deployers, including institutions, are required to perform data collection to allow effective implementation of PMS. This task should be performed at an institutional level (e.g., through a semi-automated system managed by dedicated personnel) rather than rely on voluntary action by single physicians.

Votes (57% in favor, no consensus)

Likert	Votes (n)
1	0
2	3
3	3
4	2
5	6

Statement: The deployer's responsibility for PMS is limited to notifying the provider of any issue encountered during clinical use of the AI Medical device, without bearing responsibility for how the provider will use or respond to that information.

Votes (71% in favor, no consensus)

Likert	Votes (n)
1	0
2	2
3	2
4	5
5	5

Statement: Deployers of AI medical devices should inform the referring clinician colleague and/or patients about AI-related issues and their resolution, to ensure that the patient is ultimately provided with adequate information. Ideally, foreseeable issues should also be accounted for within the informed consent process when AI medical devices are employed.

Votes (50% in favor, no consensus)

Likert	Votes (n)
1	0
2	2
3	5
4	5
5	2

Statement: The PMS should include a platform to ensure accessibility of continuously collected performance data to deployers, allowing both monitoring by medical imaging practitioners and ad hoc reporting of relevant events, as required by PMCF.

Votes (100% in favor, achieved consensus)

Likert	Votes (n)
1	0
2	0
3	0
4	5
5	9

Statement: All deployers of a given AI medical device should be able to request access to aggregate data regarding PMS and system performance, in a form compliant with current data protection regulations.

Votes (100% in favor, achieved consensus)

Likert	Votes (n)
1	0
2	0
3	0
4	4
5	10

Statement: Upon deployment of a new AI medical device, the PMS system must be made available at the same time. If a new function is added to a pre-existing device, the PMS must be contextually updated to incorporate any new information necessary.

Votes (100% in favor, achieved consensus)

Likert	Votes (n)
1	0
2	0
3	0
4	3
5	11

Statement: Beyond continuous monitoring, periodic reviews (e.g., every 6-12 months) of PMS data must be performed by providers and presented to deployers, to allow informed ongoing use of the medical device and facilitate timely detection of performance degradation.

Votes (93% in favor, achieved consensus)

Likert	Votes (n)
1	0
2	0
3	1
4	7
5	6

Statement: Some use cases (e.g., image reconstruction) may require the collection of additional data (e.g., non-AI reconstructed images) to allow periodic performance assessment of the AI medical device.

Votes (79% in favor, achieved consensus)

Likert	Votes (n)
1	1
2	0
3	2
4	3
5	8

Statement: To allow effective PMS, providers must provide the AI medical device baseline accuracy metrics, including uncertainty measures (e.g., 95% confidence intervals). These should be clearly visible on the PMS platform to deployers in order to facilitate detection of underperformance or other issues.

Votes (93% in favor, achieved consensus)

Likert	Votes (n)
1	0
2	0
3	1
4	4
5	9

Statement: The PMS platform should allow user feedback by deployers, including for PMCF purposes. This feedback should also be accessible to other deployers, through the same platform.

Votes (50% in favor, no consensus)

Likert	Votes (n)
1	0
2	2
3	5
4	2
5	5

Statement: The ESR advocates for interoperable PMS standards, easing the use of shared platforms for AI medical devices from multiple providers. This practice would greatly increase the accessibility and manageability of PMS by providers and deployers alike, as the number of AI medical devices and their adoption in clinical practice is expected to increase over the years.

Votes (100% in favor, achieved consensus)

Likert	Votes (n)
1	0
2	0
3	0
4	3
5	11

Statement: When AI medical systems are provided through shared platforms, the integration and interoperability of PMS tools should be the responsibility of AI providers (rather than healthcare institutions), to further incentivise them to prioritise and maintain effective PMS infrastructures in place.

Votes (57% in favor, no consensus)

Likert	Votes (n)
1	0
2	3
3	3
4	2
5	6

Delphi Round 2

Statement: The data collection which may be performed by deployers of AI medical devices should be implemented at an institutional level (e.g., through a semi-automated system managed by dedicated personnel within a department/hospital) rather than rely on voluntary action by single physicians.

Votes (93% in favor, achieved consensus)

Likert	Votes (n)
1	0
2	1
3	0
4	3
5	10

Statement: While the deployer has a responsibility to notify the provider of any issue encountered during clinical use of the AI Medical device, for PMS purposes, the provider is solely responsible for if/how this information is acted upon (e.g., adjusting the medical device's calibration, retraining a diagnostic model).

Votes (57% in favor, no consensus)

Likert	Votes (n)
1	0
2	2
3	4
4	3
5	5

Statement: Deployers of AI medical devices should consider informing the referring clinician colleague and/or patients about AI-related issues and their resolution, if warranted by the gravity and/or clinical relevance of the event (e.g., an incident worthy of reporting according to PMCF regulations).

Votes (93% in favor, achieved consensus)

Likert	Votes (n)
1	0
2	0
3	1
4	3
5	10

Statement: To facilitate compliance by deployers to PMCF duties, it would be preferable for the platform employed for PMS to also allow user feedback to be recorded. This information should also be accessible to other physicians within the same institution, through the same platform, to facilitate local awareness of critical issues with an AI medical device.

Votes (93% in favor, achieved consensus)

Likert	Votes (n)
1	0
2	1
3	0
4	5
5	8

Statement: Providers may employ shared software platforms to optimize access to multiple AI medical devices. In this case, the use of interoperable PMS standards is recommended to facilitate aggregation of data from all AI medical devices. For example, access through a unified user interface at the software platform level to monitor all devices delivered through said platform would be preferable rather than siloed PMS systems within each medical device's dedicated interface.

Votes (100% in favor, achieved consensus)

Likert	Votes (n)
1	0
2	0
3	0
4	3
5	11

Supplementary Fig S1. Bar plots presenting the voting results from the Delphi process in rounds 1 and 2. Only items that did not reach a consensus were included in the second round of voting. Please note that additional details on the Delphi process and its results are available in the supplementary materials.

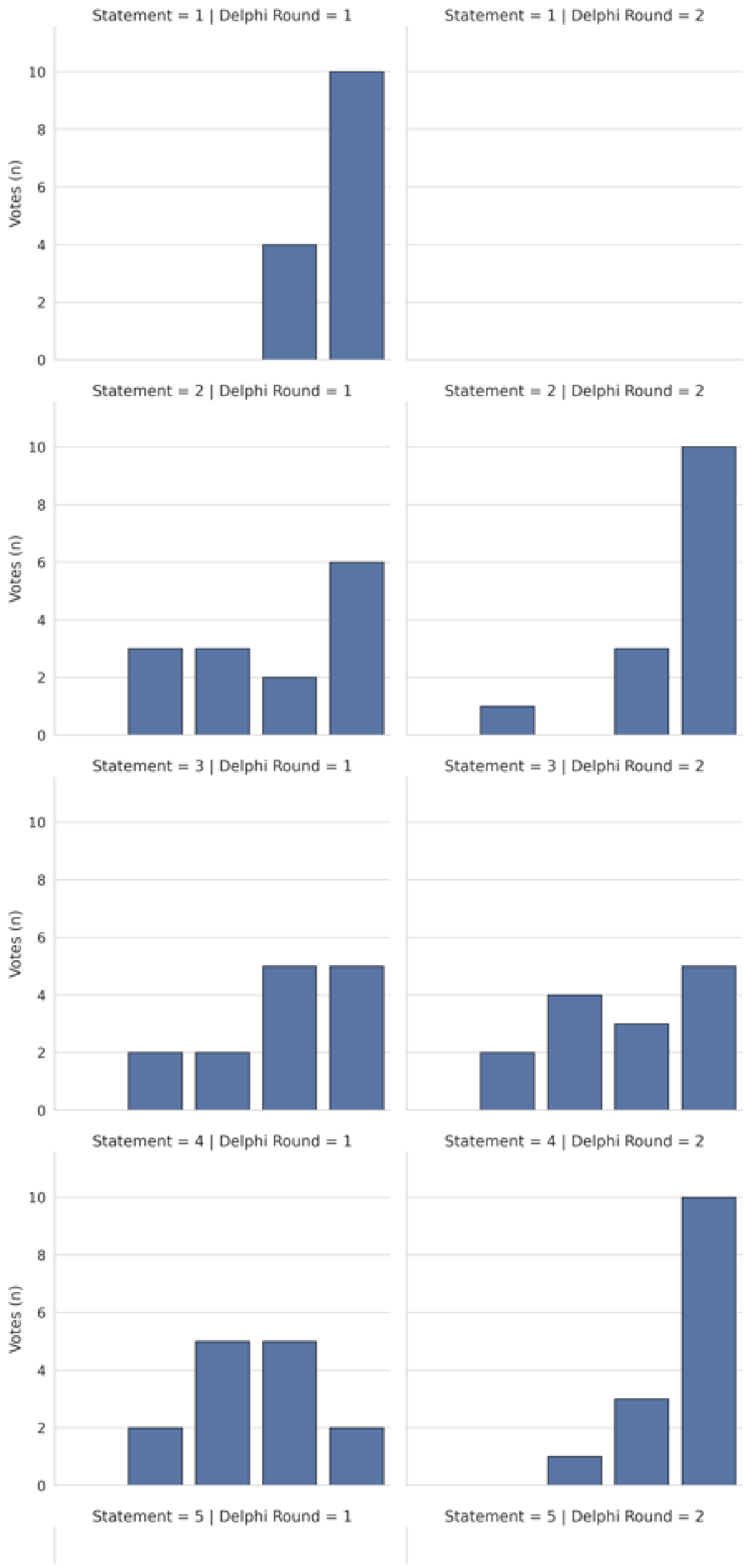
Statement = 1 | Delphi Round = 1
Statement = 1 | Delphi Round = 2
Statement = 2 | Delphi Round = 1
Statement = 2 | Delphi Round = 2
Statement = 3 | Delphi Round = 1
Statement = 3 | Delphi Round = 2
Statement = 4 | Delphi Round = 1
Statement = 4 | Delphi Round = 2
Statement = 5 | Delphi Round = 1
Statement = 5 | Delphi Round = 2
Votes (n)
0
2
4
6
8
10

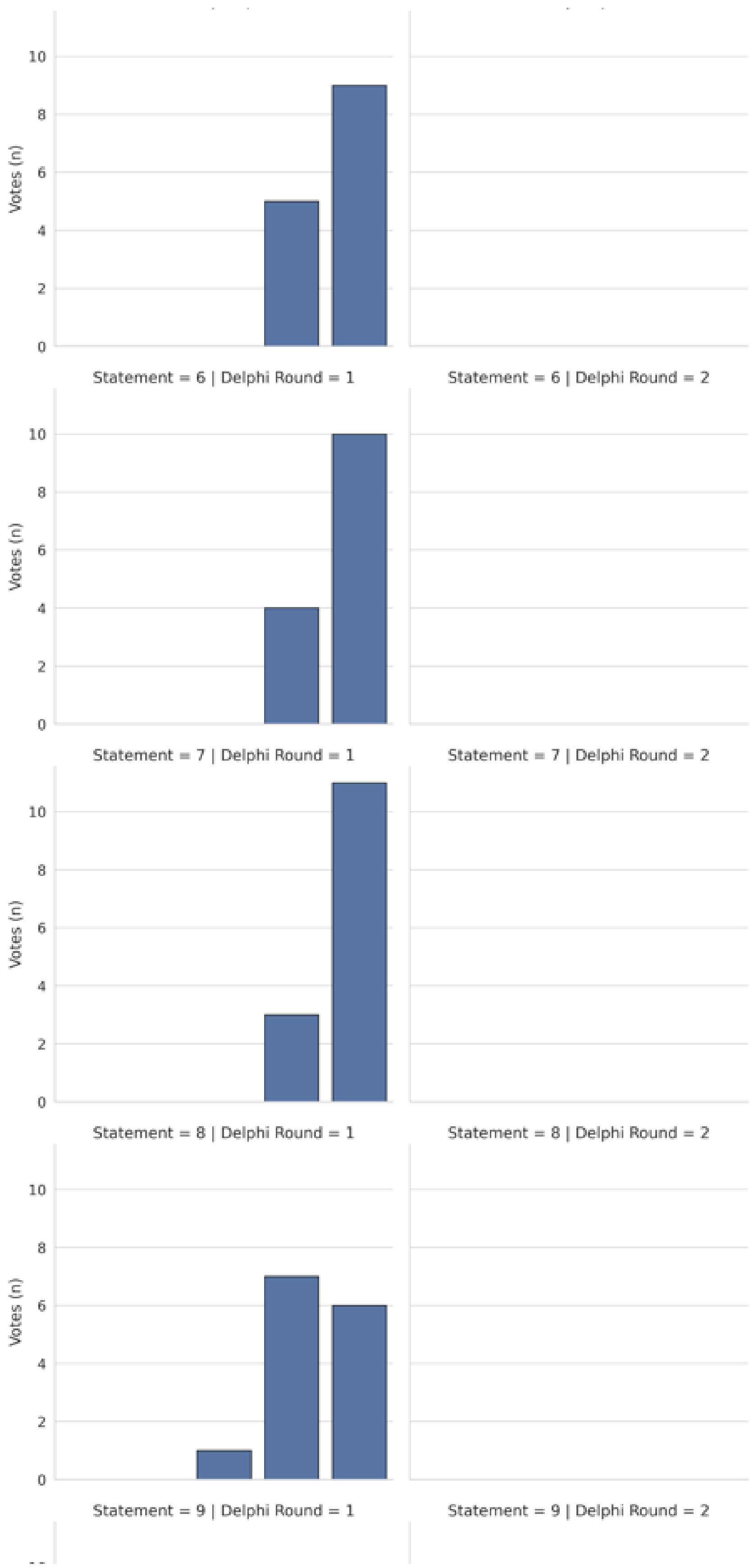

Votes (n)
0
2
4
6
8
10
Statement = 6 | Delphi Round = 1
Statement = 6 | Delphi Round = 2
Statement = 7 | Delphi Round = 1
Statement = 7 | Delphi Round = 2
Statement = 8 | Delphi Round = 1
Statement = 8 | Delphi Round = 2
Statement = 9 | Delphi Round = 1
Statement = 9 | Delphi Round = 2

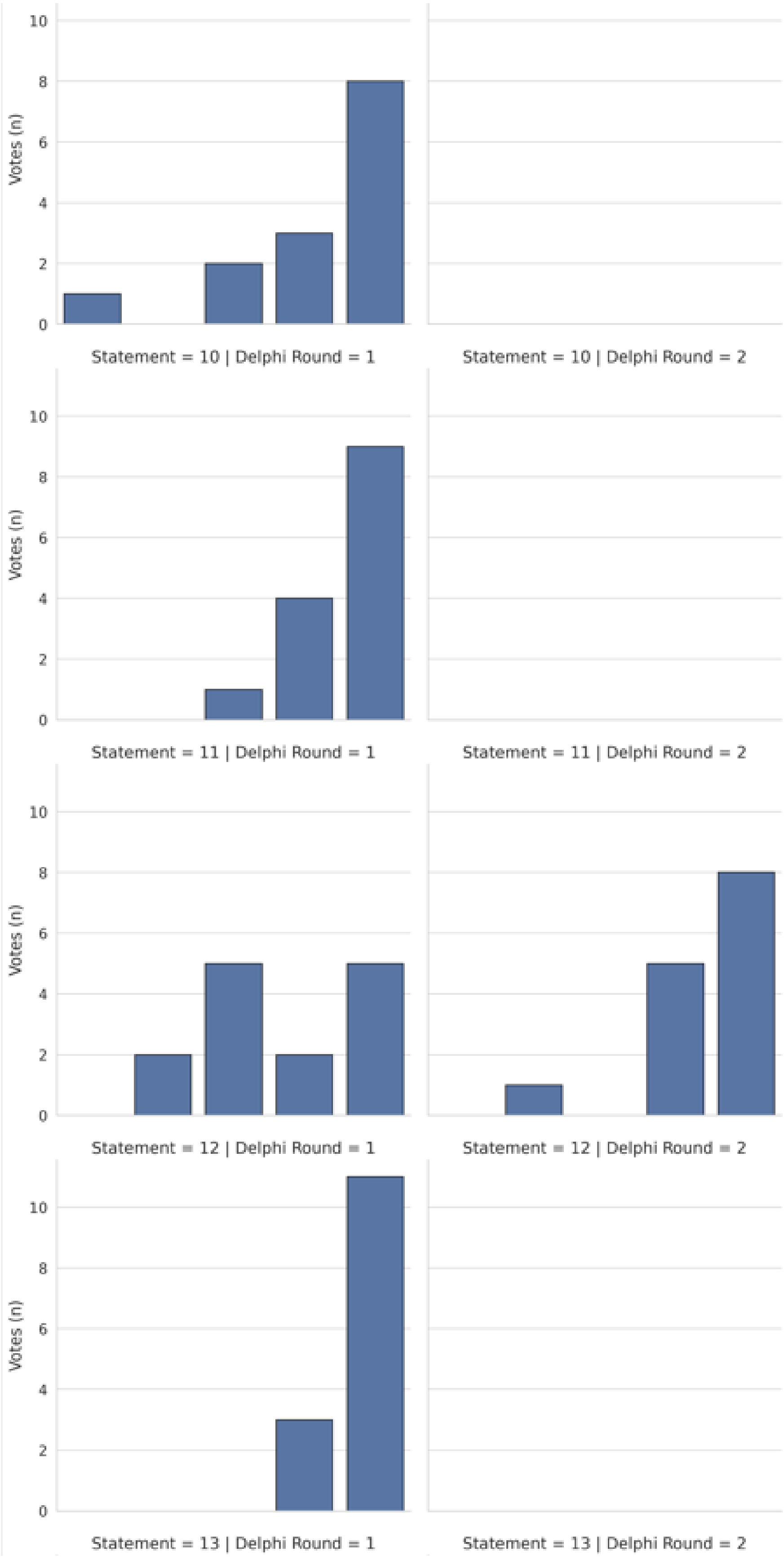
Votes (n)
Statement = 10 | Delphi Round = 1
Statement = 10 | Delphi Round = 2
Statement = 11 | Delphi Round = 1
Statement = 11 | Delphi Round = 2
Statement = 12 | Delphi Round = 1
Statement = 12 | Delphi Round = 2
Statement = 13 | Delphi Round = 1
Statement = 13 | Delphi Round = 2

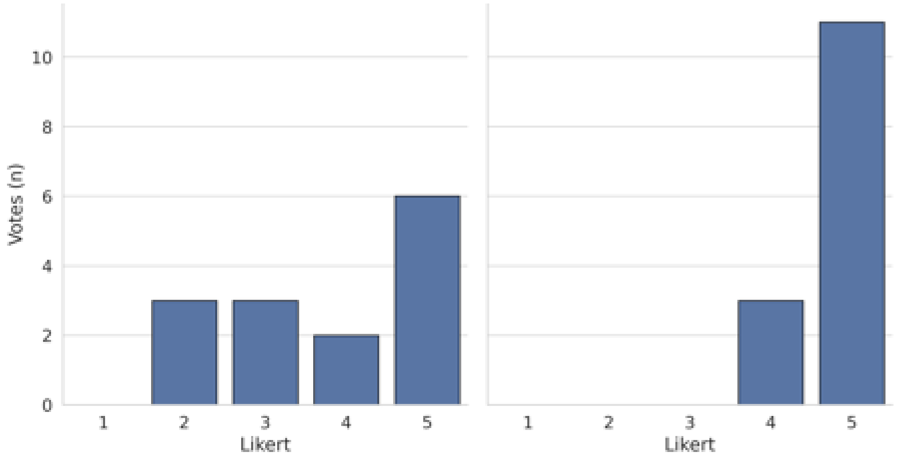
Votes (n)
10
8
6
4
2
0
1
2
3
4
5
Likert
1
2
3
4
5
Likert
